# Ethnic disparities in fertility and its determinants in Nigeria

**DOI:** 10.1186/s40738-019-0055-y

**Published:** 2019-03-30

**Authors:** Ayo Stephen Adebowale

**Affiliations:** 1 Department of Epidemiology and Medical Statistics, Faculty of Public Health, University of Ibadan, Ibadan, Nigeria; 2 Centre for Actuarial Research (CARe), University of Cape Town, Cape Town, South Africa

**Keywords:** Fertility, Ethnicity, Parity, Childbearing, Nigeria

## Abstract

**Background:**

High fertility rate has been consistently reported in Nigeria. The three major ethnic groups in Nigeria, Hausa/Fulani, Igbo, and Yoruba have different socio-cultural identities particularly those that relate to fertility but fertility index is often reported at the national level. This paper examined ethnic differences in fertility and identified its determinants in Nigeria.

**Method:**

This cross-sectional design study focused on 23,140 women aged 15–49 years. Fertility was measured from information on the full birth history of women of reproductive age. Fertility was assessed using descriptive statistics, parity progression ratio(PPR) and negative binomial model (α = 0.05).

**Results:**

The total fertility rate was 8.02, 4.91 and 4.43 among women in Hausa/Fulani, Igbo and Yoruba ethnic group respectively. The proportion of women with ≥5 children was highest among the Hausa/Fulani (40%), followed by Igbo (21.6%) and Yoruba (17.5%). For women aged 45–49 years; the PPR was highest among Hausa/Fulani while Igbo and Yoruba exhibited a similar pattern. The mean fertility was 1.725(C.I = 1.661–1.792, *p* < 0.001) times higher among Hausa/Fulani than Yoruba women, but Igbo and Yoruba women exhibited a similar pattern. Controlling for other factors barely changes this pattern.

**Conclusion:**

Variation existed in fertility across the main ethnic groups in Nigeria, but highest among Hausa/Fulani. Fertility reduction strategies that target improvement in women’s education will reduce the fertility rate in Nigeria, particularly among Hausa/Fulani women. Ethnicity is important in fertility reduction strategies in Nigeria.

## Introduction

**Fig. 1:**
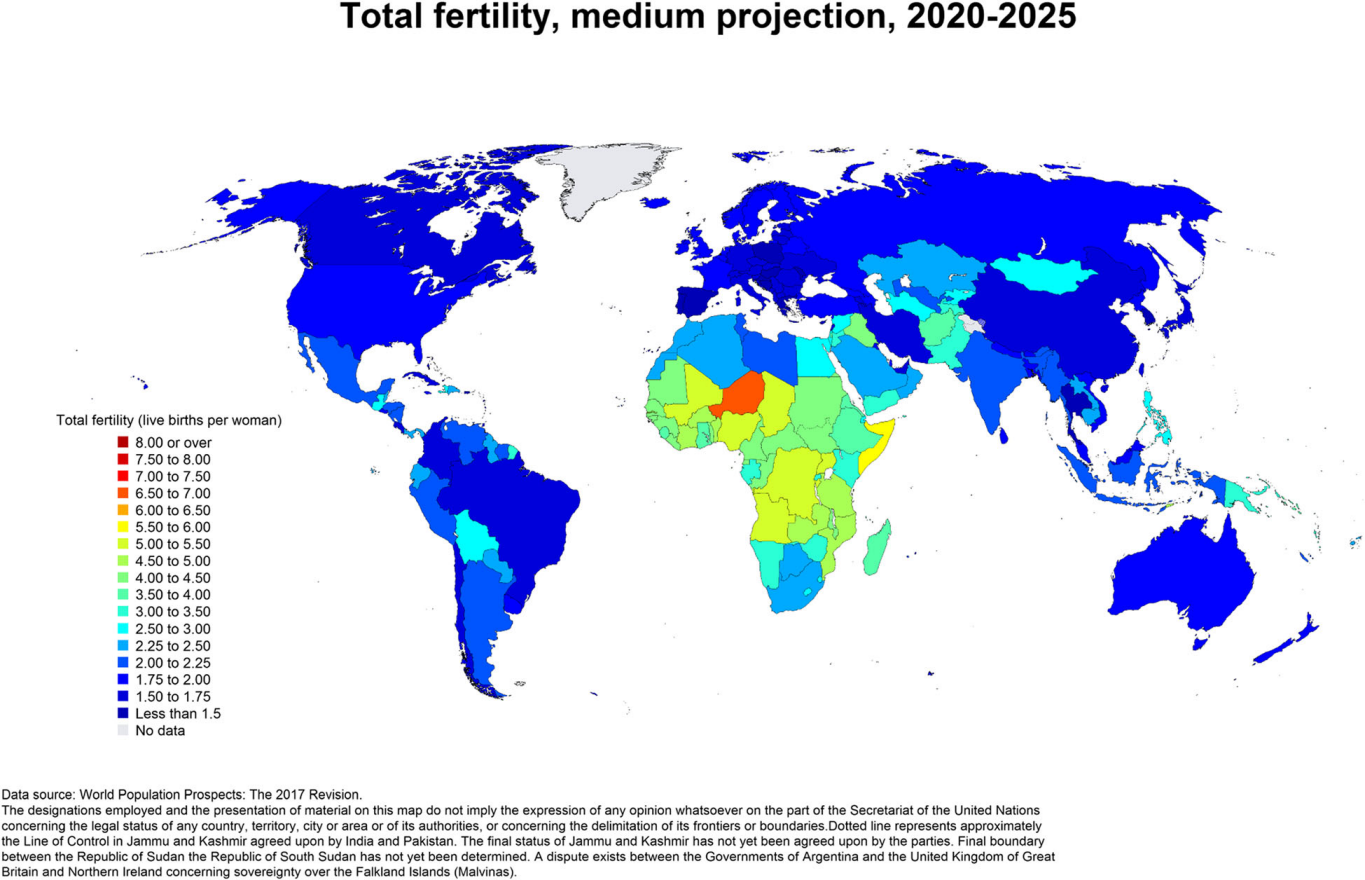
Total fertility, medium projection, 2020–2025

Fertility is one of the prime determinants of population dynamics and among the key indices for measuring the development of any nation [1]. Regardless of the poor demographic data quality in Nigeria, both indirect and direct estimates have consistently shown a high fertility level in Nigeria in the past three decades [2, 3]. In 1990, the Total Fertility Rate (TFR) was 6.01 and reduced to 5.5 in 2013 [2, 3]. Nigeria is currently the 7^th^most populous countries worldwide and by 2050 projection, the country will be the 4th most populous country globally [4]. The consistent reporting and future projection of high fertility in Nigeria, show that the country is at the first stage of demographic transition (Fig. 1). This may have deleterious implication for the country’s socio-economic advancement [5, 6] and if this persists, there is the likelihood that Nigeria might fail to meet some of the Sustainable Development Goals (SDGs). Due to the large population size of Nigeria, the country’s fertility situation is an important demographic index to be reckoned with if the goal of reducing global population growth is to be achieved. In response to the persistent high population growth rate in Nigeria, the first National Policy on Population for Development was developed in 1988 and reviewed in 2004 [7, 8]. The policy emphasizes that population factors, socio-economic development, and environmental issues are interconnected and crucial to the realization of sustainable development in Nigeria. Unfortunately, 10 years after the enactment of the policy, the TFR had reduced by only 0.2 children while the population growth rate barely changed [8].

Fertility differentials have been widely reported across socio-economic classes in Nigeria [9–11], but ethnicity is likely to be one of the important factors due to individual attachment to his/her ethnic domain. In Nigeria, individuals are geographically concentrated mainly according to the environment, ways of life and by their ethnic groups. In this country, a person’s primary loyalty to some extent is to his family, lineage and ethnic group; his allegiance to national identity is often weak [12, 13]. In spite of the heterogeneous nature of Nigeria, fertility data is still commonly analyzed based on national delineations like geopolitical zones, states, etc. for political reasons. However, both diffusion and ideational theories of fertility decline show that fertility behavior might be similar in ethnolinguistic groups. Therefore, there is the possibility that some ethnic groups are experiencing fertility changes that are being ignored in national estimates. In this context, it is imaginable that there might be some ethnic groups undergoing more rapid transition, whereas studies tend to be at the national level [14]. Demographers and population experts [13, 14] have argued that reporting fertility at the national level has a tendency to wrongly position the fertility situation of some groups on demographic transition table. This study was conducted among the three major ethnic groups in Nigeria. These are; Hausa/Fulani, Igbo, and Yoruba.

The people from Hausa/Fulani ethnic origin reside predominantly in the Northern part of Nigeria, while the Igbo and Yoruba dominate the South [15]. People from each of these tribes are found in any part of the country as a minority group. Fertility has been consistently reported to be higher in the regions dominated by the Hausa/Fulani ethnic group than those where the majority are either Yoruba or Igbo [2, 16, 17]. The use of modern contraception and female school enrolment are both higher in the Southern than the Northern part of Nigeria [2]. The main religion of Hausa/Fulani tribe is Islam and for Igbo, it is Christianity while among the Yoruba, the population is divided more or less equally between Christianity and Islam [15, 18]. Early marriage and childbearing are more pronounced among Hausa/Fulani ethnic group than the Igbo and Yoruba [2, 19]. The three groups differ in terms of cultural heritage and uphold to a greater extent these identities irrespective of their socio-economic status and location within the country.

The socio-economic characteristics described above have been previously identified as important predictors of fertility in Nigeria [9–11], but the questions that remain unanswered are; Is the fertility level more homogeneous across ethnic groups than at national level? Is the state of fertility transition the same in the three main ethnic groups? Do national data hide sub-groups for whom the fertility transition is more advanced? Therefore, the objectives of this study are to; determine the level of fertility in the three major ethnic groups in Nigeria, assess the parity progression ratio, determine an association between sociodemographic factors and fertility in each ethnic group and examine whether ethnicity is a predictor of fertility in the midst of other factors. The objectives were conceptualized in order to improve knowledge on which of the main ethnic groups in Nigeria contributes more to the national fertility level. This paper provides information and findings that are relevant to policymakers and programs in Nigeria and demonstrates non-uniformity of demographic shifts and fertility patterns in a large populous country.

## Methods

### Study and sampling design

A cross-sectional population-based study which involved secondary data analyses of the weighted sample of 2013 Nigeria Demographic Health and Survey (NDHS) was used. The sample was selected using a stratified 3-stage cluster design consisting of 904 clusters, 372 in urban areas and 532 in rural areas. A representative sample of 40,680 households was selected for the survey and a fixed sample take of 45 households was selected per cluster. However, in the current study, a sub-sample of 23,140 women which comprises of 12,409 Hausa/Fulani, 5557 Igbo and 5174 Yoruba were used. All women of reproductive age (15–49 years) irrespective of whether they have had at least a child or not were included in the study. Every woman with missing information on fertility was excluded from the analysis. At the point of data collection, the data originators excluded women who were seriously ill or mentally imbalance from the interview.

### Variable description

The dependent variable was fertility measured by children ever born (V201). This variable was derived from the detailed birth histories collected from women. The variable was re-categorized into the following groups; 0, 1–2, 3–4 and 5+ to examine the percentage of women in each tribe who belong to a specific class of childbearing. The main independent variable was ethnicity. In the questionnaire for the study, a question was asked on the ethnic group the respondent belongs (what is your ethnic group?). The question was left as open ended and as such, many ethnic groups were reported in the original data, but the variable (V131) was however re-coded by sieving out other tribes leaving only the three largest, Hausa/Fulani, Igbo and Yoruba in this study. To avoid the effect of collinearity in the regression model used, the highly correlated predictors were removed from the model. In the case where two or more factors have high variance inflated factor, one was removed from the model. Categorical principal component analysis was further used to reduce the number of predictors to a set of uncorrelated factors thus retaining the independent variables below;

**Table:** 

Variables	Question	Operationalization	DHS Code	Re-coded as
Age	How old were you on your last birthday?	Reported in years	V012	15–24; 25–34; 35–44; 45–49
Age at first birth	Age of respondent at 1st birth	Reported in years	V212	< 18 years, ≥18 years
Fertility preference	Ideal number of children	Reported in number	V613	< 5, 5+
Sex preference	Ideal number of boys, ideal number of girls	Reported in number Created as a proxy from two variables by subtracting V628 from V627. Negative or Positive values signifies sex preference and 0 no preference	V627, V628	No, Yes
Residence	Type of place of residence	Urban, Rural	V024	
Marital status	Marital status	Never in union, Currently in union/living with a man, Formerly in union/living with a man	V502	Never married, Ever married
Education	What is the highest level of school you attended?	None, Primary, Secondary, Higher	V106	
Religion	What is your religion?	Catholic, Other Christians, Islam, Traditionalist, Others	V130	Christian, Islam, Others
Wealth index	Different questions were asked based on the availability of a set of items	Wealth index factor score (5 decimals)	V191	Poor, Middle, Rich
Ever used contraceptive method	Ever used anything or tried to delay or avoid getting pregnant	No, Yes Used outside calendar, Yes Used in the calendar	V302A	No, Yes

The computation of wealth index was based on information received on ownership of the household’s consumable goods, dwelling characteristics, drinking water source, toilet facilities, and other characteristics that are related to household’s socioeconomic status. The assets were independently assigned a weight (factor score) generated with the use of principal component analysis (PCA). The obtained asset scores were standardized. A score was assigned to each asset and a total score was obtained for each household. The data originator [2] had generated the score for each respondent as indicated in the individual record data (V191). The aggregate score was therefore classified into three categories as poor, middle, and richest.

### Descriptive analyses

The data were weighted due to complex sampling procedures used during the data collection. In order to provide a 3-year trend in ASFR and TFR, the direct method based [20] on full birth histories of women of reproductive age was used to estimate TFR for the year 2010, 2011, 2012 and 2010–2012. In providing the TFR estimates, the individual record in which the unit of analysis is the woman and child record datasets were used. The individual record was used to estimate the denominator of the fertility rates based on the information on the month and year of each woman’s birth, derived from a century-month code (CMC). Detailed procedures involved in providing the fertility rates can be found on pages 111–113 of the Tools for Demographic Estimation which is available at IUSSP website [20]. The association between children ever born (CEB) and socio-demographic classes was examined using analysis of variance (ANOVA).

The TFR and mean CEB among women aged 45–49 years were compared for the evaluation of long-term change in fertility. The mean CEB among women aged 45–49 years is an indication of average completed fertility for women who commenced childbearing three decades prior to the survey. If fertility is stable over time in a population, there would be a similarity in TFR and the mean number of CEB by women age 45–49. However, for declining fertility levels, lower TFR than the mean number of CEB is the case. The number of CEB to all women of reproductive age is useful for observing primary infertility level and variation in family size across age groups. The parity progression ratio (PPR) was determined based on the designed procedure in the tool for demographic estimation (page 70) [20];

### Multivariate analysis

A negative binomial regression model was fitted for the data. The nature of fertility data for this study informed the use of the model. The dependent variable exhibited a skewed distribution with mean strikingly lower than the variance termed as over dispersion. Over dispersion thus occurs as a result of neglected unobserved heterogeneity.

The conditional variance of the negative binomial distribution surpasses its conditional mean and this makes it suitable for the analysis of this data.

The multivariate analysis was conducted to examine the relationship between fertility and ethnicity and more importantly to identify the predictors of fertility in each of the tribes. Further analysis involved the pooling of all the data for the three ethnic groups together to examine how ethnicity relates to fertility. In this instance, three models were generated. The first model involved only the ethnicity as the explanatory variable, while the second included the demographic variables. In the third model, both direct and indirect factors were included in the model. The selection of the variables into the regression model was based on Bongaarts theoretical model [21] as earlier propagated by Davis and colleague [22]. The direct variables used in the model as found in the DHS data were used in the second model. However, contraceptive use was not well captured in the survey as information on ever-used of contraceptive was only available and this does not depict the level of use. So, this variable was included among socioeconomic factors in the third model. Data were analysed at 5.0% level of significance. Akaike information criterion was used to determine the goodness of fit of the models and the preferred model is the one with the minimum AIC value. Model effect was also tested in order to know if each term in the model has any effect. Terms with significance values less than 0.05 have some discernible effect.

### Ethical consideration

Formal approval to use data-set for this study was obtained from the DHS program. The ethical approval was granted by the Nigeria National Ethics Committee (NHREC/2008/07). Informed consent was obtained from the respondents, and they were assured of the confidentiality and anonymity of the information they provided.

## Results

The characteristics of the women in the sample are shown in Table 1. About 53.6% of the respondents were women from Hausa/Fulani ethnic background, while 24.0 and 22.4% belong to Igbo and Yoruba ethnic group respectively. The mean age in years of women in Igbo (29.4 ± 9.7) and Yoruba (29.5 ± 9.6) ethnic groups was similar but slightly higher than that of Hausa/Fulani women (28.5 ± 9.7). The percentage distribution of Hausa/Fulani women reduces with increasing wealth, where 67.1% constituted the poor women and 17.7%, the rich. The converse distribution of wealth was observed among the Igbo and Yoruba women. In all the tribes, a higher proportion of the women said they don’t have sex preference, but sex preference was more prominent among the Igbo women than Yoruba and Hausa/Fulani. About 45, 11.9 and 10.1% of Hausa/Fulani, Igbo and Yoruba women had their first birth at ages below 18 years. Only 3.5% of Hausa/Fulani women had ever used any contraceptive method while 36.7% was reported among Igbo women, 50.2% was found among the Yoruba women. Childbearing preference for at least 5 children was majorly reported by Hausa/Fulani women (92.6%), followed by Igbo (61.7%) and the Yorubas (33.0%).

**Table 1:** Percentage distribution of women according to the three major ethnic groups in Nigeria by socio-demographic characteristics

Background Characteristics	Hausa/Fulani		Igbo		Yoruba	
	Frequency	%	Frequency	%	Frequency	%
Total	12,409	100.0	5557	100.0	5174	100.0
Age						
15–24	4743	38.2	2006	36.1	1790	34.6
25–34	3978	32.1	1818	32.7	1697	32.8
35–44	2513	20.3	1230	22.1	1245	24.1
45–49	1175	9.5	503	9.1	442	8.5
Mean(σ)	28.5(9.7)		29.4(9.7)		29.5(9.6)	
Residence						
Urban	3235	26.1	4081	73.4	4081	78.9
Rural	9174	73.9	1477	26.6	1093	21.1
Education						
No education	9331	75.2	255	4.6	215	4.1
Primary	1346	10.8	1056	19.0	947	18.3
Secondary	1533	12.4	3271	58.9	3005	58.1
Higher	199	1.6	975	17.5	1007	19.5
Religion						
Christian	94	0.8	5454	98.7	2933	56.8
Islam	12,239	99.1	16	0.3	2187	42.4
Others	18	0.1	56	1.0	42	0.8
Wealth Index						
Poor	8332	67.2	756	13.6	179	3.5
Middle	1879	15.1	1159	20.9	559	10.8
Rich	2198	17.7	3642	65.5	4436	85.7
Sex preference						
No	9026	72.7	3004	54.1	3404	65.8
Yes	3383	27.3	2554	45.9	1771	34.2
Age at first birth						
Never had birth	2540	20.5	2304	41.4	1708	33.0
< 18	5565	44.8	659	11.9	523	10.1
18+	4305	34.7	2595	46.7	2943	56.9
Median (range)	17(12–38)		21(12–43)		21(12–40)	
Ever used any contraceptive method						
No	11,972	96.5	3515	63.3	2577	49.8
Yes	438	3.5	2042	36.7	2597	50.2
Fertility Preference						
< 5	923	7.4	2128	38.3	3466	67.0
5+	11,487	92.6	3430	61.7	1708	33.0
Marital Status						
Never married	1342	10.8	2180	39.2	1629	31.5
Ever married	11,068	89.2	3377	60.8	3545	68.5

**Fig. 2:**
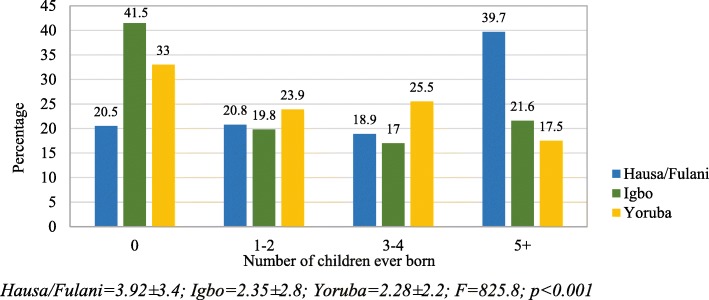
Percentage distribution of number of children ever born

In Fig. 2, the data show that 41.5% of Igbo women have never had any children and this was the highest percentage found among the three tribes. About 26% of Yoruba women had given birth to 3–4 children, compared to 18.9 and 17.0% found among Hausa/Fulani and Igbo women respectively. A wide gap was found between the Hausa/Fulani (39.7%) and other ethnic groups (Igbo: 21.6%, Yoruba: 17.5%) in terms of women who have had at least five children.

**Fig. 3:**
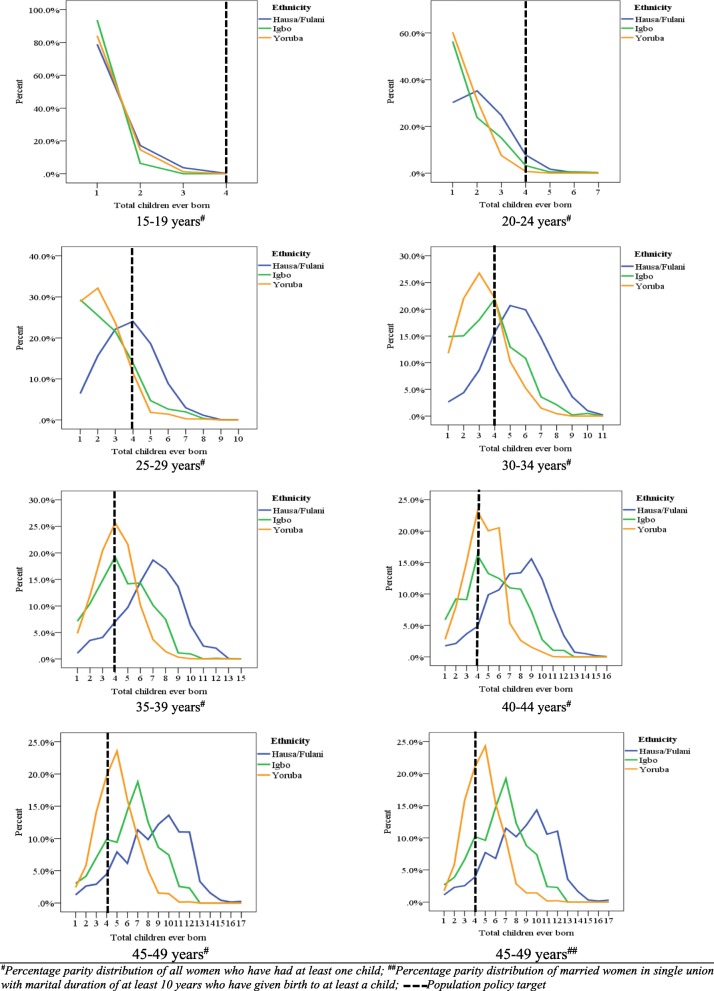
Proportion of women that have attained a specific parity

Figures 3a-h show the proportion of women in Hausa/Fulani, Igbo and Yoruba ethnic groups that have attained a specific parity standardized by age of women. The pattern of childbearing in each ethnic group was different across the age segment of women’s population. While about 80.0% of women aged 15–19 years in each ethnic groups have given birth to at least a child, few women (less than 15%) have given birth to at least two children. Among women aged 20–24 years, the highest proportion of those who have given birth to four children was found among Hausa/Fulani women, closely followed by Igbo and then Yoruba women and this pattern was found among women aged 25–29 years. The data further show that the proportion of Hausa/Fulani women who have given birth to more than four children was higher than that of Igbo and Yoruba women in the age groups, 30–34, 35–39, 40–44 and 45–49 years. There is also an indication that Igbo women aged 25–29, 30–34, 35–39, 40–44 and 45–49 years had experienced higher order births than their counterparts who are of Yoruba ethnic background.

**Fig. 4:**
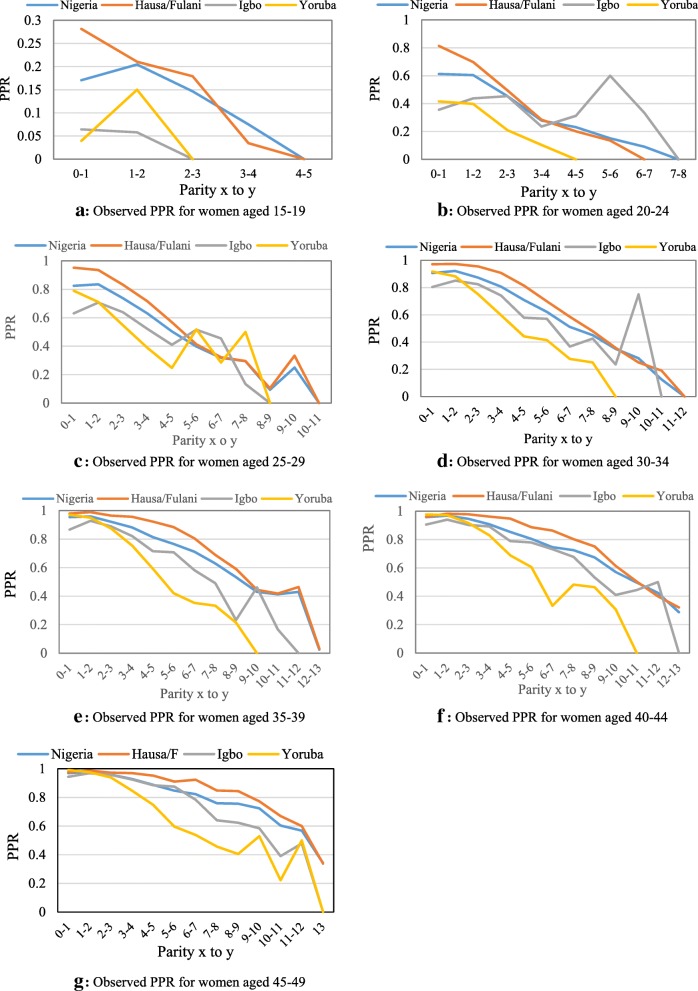
Parity progression ratio and ethnicity

Figures 4a-e depicts how the women in specific age groups move from one parity to the next higher parity according to ethnicity. In all the age groups, the data showed some similarities in the pattern of PPR between Yoruba and Igbo women while women in Hausa/Fulani ethnic group progressed at a higher rate than Yoruba and Igbo.

**Fig. 5:**
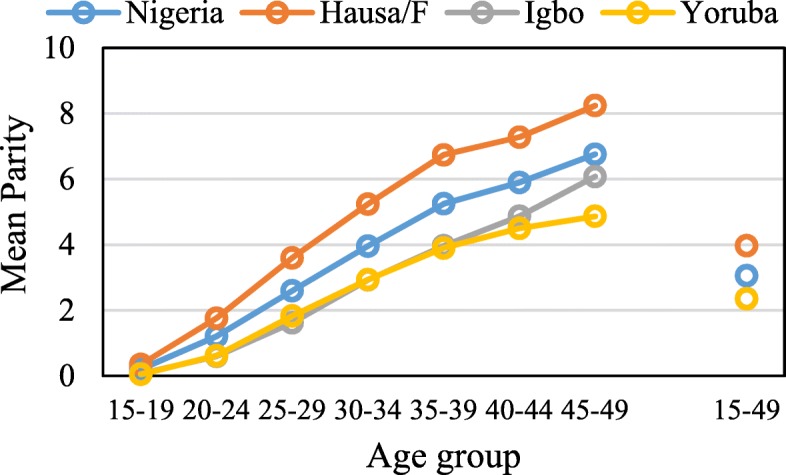
Mean parity by age group

In Fig. 5, the data show that the mean parity (φ) was higher among Hausa/Fulani women than Igbo and Yoruba women across the 5-year age groups (15–19, 20–24, …, 45–49), while Yoruba and Hausa/Fulani women aged 15–39 years exhibited similar pattern. However, among women aged 45–49 years where completed fertility is expected, the Igbo women had higher mean parity (φ = 6.08) than their counterparts who belong to Yoruba (φ = 4.87) ethnic background. Among all women who participated in the study, the Hausa/Fulani women (3.97) experienced highest mean parity, followed by Igbo (φ = 2.36) and then Yoruba women (φ = 2.35). The mean parity observed for all women aged 15–49 years in Nigeria was 3.06 while 6.76 was found for those women aged 45–49 years.

**Fig. 6:**
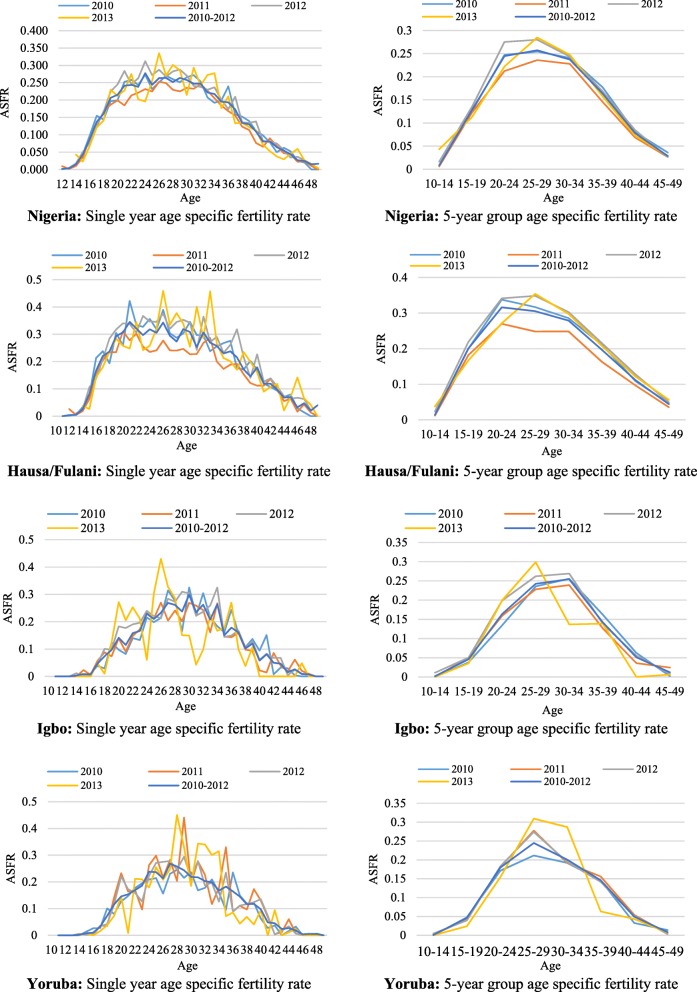
Single year and 5-year age group age specific fertility rate

The age-specific fertility rate in a single year and 5-year age group are presented in Fig. 6 across the main ethnic groups in Nigeria. The 5-year age classification smoothed out variations that are observed in single year ASFR. The pattern of age-specific fertility rate observed in the three ethnic groups was in line with the conventional dome-shaped pattern of ASFR observed for any country. The unconventional shape of the age-specific fertility rates found for the year 2013 across all the ethnic groups is an indication of incomplete data since data were only obtained for a fraction of the survey year. Among Hausa/Fulani women, the ASFR peaked at 20–24 years while it was 30–34 and 25–29 years for Igbo and Yoruba women respectively. The computed ASFR also shows that the proportion of Hausa/Fulani women who give birth at ages 45–49 years was higher than their Igbo and Yoruba age cohorts.

**Fig. 7:**
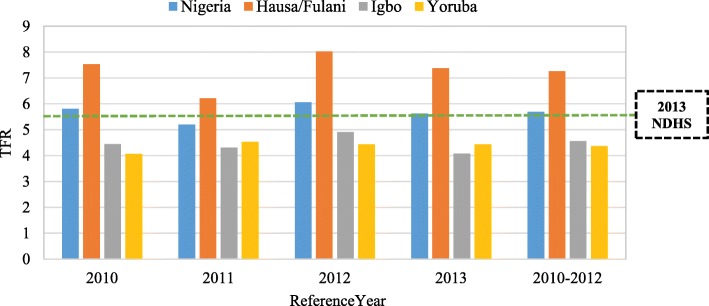
Trends in total fertility rate estimated by direct method

In Fig. 7, the data show that the TFR estimated for a 3-year period prior to the survey year (2013) was consistently highest among Hausa/Fulani women than the estimate for Igbo and Yoruba women. Despite the similarity of the pattern of TFR among Igbo and Yoruba women, the TFR obtained for Igbo women was slightly higher than that of Yoruba women in the year, 2010, 2012 and 2010–2012 but was lower in the year 2011 and 2013. From the year 2010 to 2013, where TFR was estimated, the highest TFR for Hausa/Fulani (TFR = 8.02), Igbo (TFR = 4.91) and Yoruba (TFR = 4.43) were obtained in 2012. It is also important to note that the TFR among Hausa/Fulani women was consistently higher than that of the national estimate while that of Igbo and Yoruba women was lower.

As shown in Table 2, higher mean children ever born (mCEB) was consistently found among women living in the rural areas than urban areas in each ethnic groups. It is worth noting that the mCEB by women living in the rural areas in Igbo (2.55 ± 2.8) and Yoruba (2.69 ± 2.4) ethnic groups was lower than the estimated mCEB found among Hausa/Fulani women living in urban areas (3.45 ± 3.4). The mCEB reduces with increasing level of education, household wealth and age at first birth across the three ethnic groups with an exception of women in higher and secondary education groups among Hausa/Fulani women which shows a reverse pattern. Also, the childbearing behavior of women from poor households in Igbo (3.54 ± 3.4) and Yoruba (2.99 ± 2.6) ethnic groups was similar to that of the rich women in Hausa/Fulani ethnic group (3.18 ± 3.2). In all the ethnic groups, Muslim women have higher mCEB than their Christian counterparts.

**Table 2:** Distribution of mean children ever born by background characteristics according to the three major ethnic groups in Nigeria

Background Characteristics	Hausa/Fulani		Igbo		Yoruba	
	$\overline{x}\left(\sigma \right)$	*F*-value (*p-*value)	$\overline{x}\left(\sigma \right)$	*F*-value (*p-*value)	$\overline{x}\left(\sigma \right)$	*F*-value (*p*-value)
Total	3.92(3.4)		2.35(2.8)		2.28(2.1)	
Age		6559.79***		1071.71***		2271.35***
15–24	0.98(1.2)	(< 0.001)	0.33(0.8)	(< 0.001)	0.30(0.7)	(< 0.001)
25–34	4.28(2.0)		2.18(2.1)		2.36(1.6)	
35–44	6.92(2.7)		4.37(2.7)		4.11(1.7)	
45–49	8.21(3.2)		6.08(2.9)		4.80(1.9)	
Residence		88.49***		9.95**		49.50***
Urban	3.45(3.4)	(< 0.001)	2.28(2.7)	(0.002)	2.17(2.1)	(< 0.001)
Rural	4.09(3.3)		2.55(2.8)		2.69(2.4)	
Education		362.99***		648.26***		333.86***
No education	4.39(3.4)	(< 0.001)	6.13(2.8)	(< 0.001)	4.10(2.2)	(< 0.001)
Primary	3.65(3.3)		4.51(3.1)		3.82(2.0)	
Secondary	1.55(2.4)		1.60(2.2)		1.93(2.1)	
Higher	2.31(2.6)		1.56(2.0)		1.48(1.6)	
Religion		8.67***		46.18***		10.56***
Christian	2.50(2.7)	(< 0.001)	2.31(2.8)	(< 0.001)	2.18(2.2)	(< 0.001)
Islam	3.93(3.4)		2.68(2.3)		2.43(2.1)	
Others	4.45(5.3)		5.85(2.9)		1.59(2.0)	
Wealth Index		99.54***		121.02***		28.76***
Poor	4.21(3.6)	(< 0.001)	3.54(3.4)	(< 0.001)	2.99(2.6)	(< 0.001)
Middle	3.52(3.3)		2.76(3.2)		2.78(2.5)	
Rich	3.18(3.2)		1.98(2.4)		2.19(2.1)	
Sex preference		55.89***		15.35***		7.15**
No	4.06(3.4)	(< 0.001)	2.48(2.8)	(< 0.001)	2.34(2.2)	(0.008)
Yes	3.56(3.2)		2.19(2.7)		2.17(2.2)	
Age at first birth						
No birth	^a^	361.04***	^a^	340.35***	^a^	323.89***
< 18	5.35(3.1)	(< 0.001)	5.42(2.8)	(< 0.001)	4.26(1.9)	(< 0.001)
18+	4.39(2.8)		3.66(2.3)		3.25(1.7)	
Ever used any contraceptive		32.24***		69.48***		505.73***
No	3.89(3.4)	(< 0.001)	2.12(2.8)	(< 0.001)	1.62(2.1)	(< 0.001)
Yes	4.82(2.8)		2.75(2.7)		2.93(2.1)	
Fertility Preference		346.48***		479.37***		1056.40***
< 5	1.97(2.9)	(< 0.001)	1.36(1.8)	(< 0.001)	1.65(1.8)	(< 0.001)
5+	4.08(3.3)		2.96(3.1)		3.56(2.3)	
Marital Status		246.29***		427.18***		4743.85***
Never married	0.01(0.1)	(< 0.001)	0.09(0.4)	(< 0.001)	0.05(0.3)	(< 0.001)
Ever married	4.40(3.3)		3.81(2.7)		3.30(1.9)	

***p < 0.001; **p < 0.01; ^a^:not applicable

The multivariate analysis shows that the predictors of fertility among the Hausa/Fulani women were; age, education, age at first and fertility preference and these are common predictors among the three ethnic groups. For Igbo and Yoruba ethnic groups, similar variables like age, education, sex preference, ever used any contraceptive method, age at first birth fertility preference and marital status were identified as predictors of fertility except sex preference which was not statistically significant in Yoruba ethnic group. In all the three ethnic groups, the incidence rate ratio (IRR) of fertility increases as the age group of women increases but falls consistently with the increasing level of education. For example, Hausa/Fulani women in age group 15–24, 25–34 and 35–44 years were 0.212(C.I = 0.196–0.229, *p* < 0.001), 0.515(C.I = 0.480–0.552), and 0.846(C.I = 0.786–0.910) times respectively likely to bear children than those in the age group 45–49 years and this pattern was found among Igbo and Yoruba women.

There was no significant difference between the IRR of fertility of women who had secondary education and those with higher education among Hausa/Fulani and Igbo ethnic groups, but the difference was significant among Yoruba women. However, the direction and pattern of fertility IRR were similar across the tribes with respect to the level of education. Among the Igbo ethnic group, for instance, the fertility IRR was 1.399(IRR = 1.144–1.710) and 1.369(IRR = 1.185–1.583) times higher among women that have no formal education and primary education respectively than those with higher education. Igbo women who have no sex preference compared to those who have sex preference were expected to have a rate 1.106(C.I = 1.018–1.201) times greater for fertility. Likewise, Igbo women who began childbearing at ages below 18 years compared to those who began theirs at ages ≥18 years were expected to have a greater fertility rate of 1.358(C.I = 1.229–1.501) and this was the pattern exhibited across the Yoruba and Hausa/Fulani women. Across the three tribes, women who have preference for bearing at least 5 children experienced higher fertility than those who preferred less. Lower IRR was found among the Yoruba women who have a preference for less than 5 children than those who prefer to have at least 5 children (IRR = 0.772; C.I = 0.707–0.842), however, the gap was narrower among Hausa/Fulani women than Yoruba and Igbo women.

The first model in Table 3 shows that no significant difference existed between the fertility IRR of Yoruba and Igbo women but the difference was statistically significant among the Hausa/Fulani women (IRR = 1.725; C.I = 1.661–1.792). A reduction (from 72.5 to 14.8%) in the fertility IRR of Hausa/Fulani women (IRR = 1.148; C.I = 1.021–1.292) was observed when other variables were included in the model compared to Yoruba women. Other important predictors of fertility in addition to ethnicity were; age, education, sex preference, ever used any contraceptive method, age at first, fertility preference and marital status.

**Table 3:** Predictors of fertility by background characteristics in Nigeria

Background Variables	Hausa/F	Igbo	Yoruba	All	All	All
				Model 1	Model 2	Model 3
	aIRR(C.I)	aIRR(C.I)	aIRR(C.I)	aIRR(C.I)	aIRR(C.I)	aIRR(C.I)
Age						
15–24	0.212*** (0.196, 0.229)	0.311*** (0.262, 0.370)	0.333*** (0.279, 0.399)		0.222*** (0.208, 0.236)	0.234*** (0.219, 0.250)
25–34	0.515*** (0.480, 0.552)	0.560*** (0.495, 0.633)	0.610*** (0.539, 0.691)		0.518*** (0.492, 0.546)	0.536*** (0.508, 0.566)
35–44	0.846*** (0.786, 0.910)	0.825** (0.732, 0.929)	0.905 (0.799, 1.024)		0.838*** (0.794, 0.885)	0.846*** (0.801, 0.894)
45–49	1	1	1		1	1
Ethnicity						
Hausa/F				1.725*** (1.661, 1.792)	1.398*** (1.334, 1.466)	1.148* (1.021, 1.292)
Igbo				1.043 (0.997, 1.091)	1.135*** (1.07, 1.199)	1.031 (0.925, 1.148)
Yoruba				1	1	1
Residence						
Urban	1.002 (0.932, 1.077)	0.996 (0.911, 1.089)	0.934 (0.833, 1.047)			0.998 (0.952, 1.046)
Education						
No education	1.246* (1.007, 1.541)	1.399** (1.144, 1.710)	1.348** (1.109, 1.638)			1.333*** (1.213, 1.465)
Primary	1.262* (1.015, 1.569)	1.369*** (1.185, 1.583)	1.396*** (1.220, 1.598)			1.364*** (1.250, 1.487)
Secondary	1.160 (0.931, 1.444)	1.110 (0.981, 1.255)	1.252*** (1.113, 1.409)			1.208*** (1.117, 1.307)
Higher	1	1	1			1
Religion						
Christian	0.853 (0.447, 1.630)	0.991 (0.729, 1.348)	1.014 (0.612, 1.680)			0.944 (0.746, 1.195)
Islam	0.961 (0.552, 1.671)	0.824 (0.425, 1.597)	1.032 (0.623, 1.710)			0.990 (0.779, 1.259)
Others	1	1	1			1
Wealth Index						
Poor	1.016 (0.927, 1.113)	1.055 (0.926, 1.202)	0.979 (0.785, 1.220)			1.041 (0.978, 1.109)
Middle	0.983 (0.897, 1.078)	1.007 (0.903, 1.123)	1.082 (0.940, 1.245)			1.027 (0.966, 1.092)
Rich	1	1	1			1
Sex preference						
No	1.032 (0.982, 1.085)	1.106 (1.018, 1.201)*	1.091 (0.999, 1.191)			1.057** (1.017, 1.098)
Age at first birth						
< 18	1.386*** (1.326, 1.450)	1.358*** (1.229, 1.501)	1.268*** (1.135, 1.416)		1.419*** (1.367, 1.473)	1.367*** (1.316, 1.420)
18+	1	1	1		1	1
Ever used any contraceptive						
No	0.896 (0.801, 1.003)	0.804*** (0.739, 0.874)	0.873** (0.804, 0.947)			0.855*** (0.812, 0.900)
Fertility Preference						
< 5	0.869* (0.780, 0.967)	0.749*** (0.678, 0.828)	0.772*** (0.707, 0.842)			0.807*** (0.765, 0.852)
5+	1	1	1			1
Marital Status						
Never married	1.150 (0.063, 16.095)	0.538*** (0.426, 0.679)	0.684* (0.490, 0.954)			0.660*** (0.547, 0.795)
Married	1	1	1			1
LogLL	−26,782.020	− 7943.533	− 7838.901	−54,442.465	−42,934.796	−42,598.395
AIC	53,610.039	15,933.066	15,723.802	108,890.929	85,885.591	85,246.791
Deviance (v/df)	0.105	0.092	0.074	1.074	0.108	0.098

***p < 0.001; **p < 0.01; *p < 0.05; *aIRR* adjusted incidence rate ratio, *AIC* Akaike Information Crriteria

## Discussion

Fertility determines the population size, structure, and composition of any society, while ethnicity is a social cluster of individuals that shares a unique culture, religion, language, beliefs or certain characteristics that distinguish them from their neighboring communities. Ethnicity may not be genetic but it describes people by a group different from their birth identity if they live for a long period in a new locality and they imbibe the culture, symbols, and relationships of their host community [23]. Central inquiry on the relationship between fertility and ethnicity is; how socio-cultural identities that are entrenched in ethnicity tend to influence fertility behaviors? The answer to this question is yet to be fully documented in Nigeria. This study examined fertility level and identifies its determinants in the three major ethnic groups in Nigeria.

About half of the studied women belong to the Hausa/Fulani ethnic group, while Igbo and Yoruba women shared a quarter and slightly above one-fifth respectively. The distribution of women by ethnic group found in this study reflects true ethnic composition in Nigeria [15]. It is striking that women of Hausa/Fulani ethnic origin have the bulk of its members not having formal education while only very few were found among Igbo and Yoruba women. This echoes the extent to which female education enrolment was in place among the ethnic groups and also indicates the consequence of early marriage and early childbearing tradition which is being practiced among Hausa/Fulani tribe which has been reported in previous studies [24, 25]. In this study, about one-half of Hausa/Fulani women had their first birth at ages below 18 years and this was the reason for the least median age at childbearing found among Hausa/Fulani (17 years) women compared to Igbo (21 years) and Yoruba (21 years). Similar studies supported these findings [24–26].

The study revealed that in the 3-year period (2010–2012) prior the survey year, the TFR obtained for Hausa/Fulani women was 7.26, while that of Igbo and Yoruba was 4.56 and 4.37 respectively. Thus, the estimated TFR for Hausa/Fulani ethnic group was above the national figure in Nigeria (5.5) [2] while that of Igbo and Yoruba were less. This is an indication that fertility transition has commenced among Yoruba and Igbo while Hausa/Fulani remain at the first stage of the transition. The mean number of CEB to women aged 45–49 years in the three ethnic groups under investigation was 8.2, 6.1 and 4.8 among Hausa/Fulani, Igbo and Yoruba respectively. This is approximately 0.23, 1.18 and 0.09 child more than the current TFR for Hausa/Fulani, Igbo and Yoruba women respectively, suggesting that fertility has declined over the past few decades in the three ethnic groups. Since the three ethnic groups studied are the main ethnic groups in Nigeria and they constitute the majority of Nigerians, this finding is consistent with the literature on national fertility estimate in Nigeria [2, 17].

The findings from the multivariate analysis show that the likelihood of fertility was higher among the Hausa/Fulani ethnic group than the Yoruba but no difference was observed between Igbo and Yoruba. In the literature, higher fertility has been consistently reported in the regions where the majority of the people are Hausa/Fulani than the regions where people from Yoruba or Igbo ethnic background dominate [2, 10, 17] . Similarity in the distribution of socioeconomic characteristics of women by CEB among Igbo and Yoruba might be a possible explanation for this outcome.

The study further showed that all the identified determinants of fertility among Hausa/Fulani like age, education, age at first and fertility preference were common to other ethnic groups but in addition, other factors like sex preference, ever used any contraceptive method and marital status were found among Igbo and Yoruba ethnic groups. As expected, the fertility incidence increases with age, and age at first birth. This is primarily because early commencement of childbearing predisposes women to increase the number of years of childbearing exposure within reproductive age range [27]. This pattern was similar across the three ethnic groups. Other findings in almost every society corroborated this outcome [27, 28]. Also, in this study, fertility incidence ratio reduces as the level of education increases and this situation was observed among the women in all the tribes. This is just a re-affirming of what is already known about the relationship between education and fertility [27]. However, the gap between the risk of greater fertility among women with higher education compared to those with no formal, primary and secondary education was narrower among women from Hausa/Fulani tribes compared to Igbo and Yoruba. In this context, religion is likely to be the principal reason for the Hausa/Fulani situation. Among Hausa/Fulani Muslims, it is likely that education is yet to have pronounced effect on childbearing behaviors like contraceptive use, early marriage, and early childbearing.

### Implications for policy and future research

Ethnic diversity has the tendency to enhance societal values, customs and the attainment of suitable demographic change, but must be planned for through the formulation of intercultural policies and programs development. Examining the relationship between ethnicity and fertility has a direct consequence on population policies and programmes. The goal of the National Policy on Population for Sustainable Development (NPSD) in Nigeria is to achieve a reduction in the TFR of at least 0.6 children every 5 years [8]. The fertility level by ethnic group as found in this study suggests that the Hausa/Fulani ethnic group is yet to commence fertility transition and it is likely that this ethnic group might not accomplish the target set by the NPSD.

Nigeria is currently facing the challenge of rapid population growth driven by high fertility rate. If the future generations of Nigerians must have a good living standard, the high fertility rate must be checked and the ethnic traditional customs that are inimical to fertility controls must be critically addressed through policies and programs. Otherwise, by 2050, a huge number of Nigerians will live to see more dreadful consequences of overpopulation like acute poverty, widespread unemployment, high crime rate, environmental degradation, congestion than what is being witnessed currently. In the quest to reduce the level of fertility in Nigeria, research that targets men will be a welcome development towards the accomplishment of this goal.

### Limitation

The data may be susceptible to bias often resulted from misclassification of birth timing due to recall bias. In the African context, there is the likelihood of omitting dead children from the full birth history list, thus there might be under-estimation of fertility level. Also, distortions as a result of displacement and omission of births might lead to underestimation of fertility. However, the data originator ensures that these biases were minimized at the points of the study design and data collection. It is also necessary to note that some covariates at the date of the interview may not have applied to the time the birth of a specific child took place. Due to the nature of the data (secondary) used for this study, there is an extent to which some theoretical frameworks can work effectively in the building of the model. However, the choice of the variables for this study was based on the availability of the variables in the data as reconciled with those in the existing theoretical frameworks.

## Conclusion

Fertility remains high in all the three main ethnic groups in Nigeria, but more prominent among Hausa/Fulani women. The Igbo and Yoruba exhibited similar fertility pattern. Fertility rates and behavior were more homogeneous within the three ethnic groups than national groups and the state of fertility transition is not the same across the groups. While the Igbo and Yoruba ethnic groups are currently undergoing fertility transition, such is yet to commence among Hausa/Fulani. Thus, the national data hide the Igbo and Yoruba sub-groups for whom the fertility transition is more advanced. Although, there were differences between the predictors of fertility among the three tribes, the common predictors of fertility among the tribes were; age, education, age at first and fertility preference. Improving female education and school enrollment will be an important fertility reduction intervention in Nigeria, particularly among Hausa/Fulani women.
